# Clinical findings of 40 patients with nocardiosis: A retrospective analysis in a tertiary hospital

**DOI:** 10.3892/etm.2014.1715

**Published:** 2014-05-14

**Authors:** MEIFANG YANG, MIN XU, WEI WEI, HAINV GAO, XUAN ZHANG, HONG ZHAO, JIANHUA HU, HUIHUI DONG, LICHEN XU, LANJUAN LI

**Affiliations:** 1 State Key Laboratory for Diagnosis and Treatment of Infectious Diseases, The First Affiliated Hospital, School of Medicine, Zhejiang University, Hangzhou, Zhejiang 310003, P.R. China; 2 Collaborative Innovation Center for Diagnosis and Treatment of Infectious Diseases, Hangzhou, Zhejiang 310058, P.R. China

**Keywords:** *Nocardia*, corticosteroids, opportunistic disease, pulmonary nocardiosis, brain abscess

## Abstract

To the best of our knowledge, no Chinese case studies concerning *Nocardia* infection have been published to date. Therefore, the present study aimed to retrospectively evaluate the risk factors, clinical features, imaging results, laboratory abnormalities, treatments and outcomes of nocardiosis in a Chinese tertiary hospital. Data collected from patients with laboratory-confirmed nocardiosis were retrospectively analyzed. A total of 40 patients who had a positive culture of *Nocardia* were included. The median time between the onset of symptoms and diagnosis was 42 days. Underlying diseases were identified in 72.5% of the patients of which diabetes was the most common (32.5%). The most important risk factor was corticosteroid administration. Fever and cough were common clinical symptoms. The pleuropulmonary (85%) were the most frequently involved sites and the disseminated disease rate was 30.0%. Frequent chest computed tomography scans revealed the presence of airspace opacities, nodules and masses, in addition to cavitary lesions that were particularly common among the study group. Brain images revealed lesions associated with abscesses. The majority of the patients (71.1%) were treated with trimethoprim sulfamethoxazole alone or in combination with other drugs. The in-hospital mortality rate was 15.0%. Disseminated disease, immunocompromised patients, an older age, brain involvement and concomitant infections were associated with a poor prognosis. Nocardiosis is an uncommon but emerging disease. The present study reports the first case series on nocardiosis from China and provides important information on the clinical features and risk factors of nocardiosis. Early recognition of the disease and the initiation of appropriate treatment are essential for a good prognosis.

## Introduction

*Nocardia* are gram-positive, aerobic, slow-growing pathogens that are widespread in the environment and are found in soil, vegetation, other organic matter, fresh water and salt water (1). In humans, they are mainly acquired through direct inhalation or inoculation. *Nocardia* are rare opportunistic pathogens that particularly affect patients who are on immunosuppressive therapy or chemotherapy, post-transplant patients, and patients with human immunodeficiency virus (HIV) infection. There are few studies and case reports available on nocardiosis (2–9). Thus, there are no optimal antimicrobial regimens available to date. Furthermore, to the best of our knowledge, no large studies or case series have been reported from China. Hence, the current retrospective study was carried out to focus on the clinical characteristics of Chinese patients with a confirmed diagnosis of nocardiosis.

## Materials and methods

### Study design

The medical records of patients with a confirmed diagnosis of nocardiosis, who were hospitalized at the First Affiliated Hospital of Zhejiang University (Hangzhou, China) between January 2000 and May 2013, were retrospectively reviewed. The condition of all patients was confirmed as nocardiosis through the presence of a positive culture of *Nocardia*. The possible risk factors, clinical features, laboratory abnormalities, treatments and outcomes of these patients were analyzed by the review of all medical, epidemiological, clinical and laboratory data by two infectious disease experts. Radiological results were obtained from the review of radiographs by an independent radiologist, who was blinded with regards to the clinical findings. The ethics committee of the hospital reviewed and approved the study protocol. Written informed consent was obtained from all participants.

Specimens were inoculated in normal blood using nonselective media. *Nocardia* species identification and susceptibility tests were carried out only upon special request and therefore the data on *Nocardia* assessments were available for only a limited number of patients.

### Statistical analysis

All statistical analyses were performed using SPSS software, version 17.0 (SPSS, Inc., Chicago, IL, USA). Continuous variables were expressed as medians with interquartile ranges. The percentages of patients in each category were calculated for categorical variables.

## Results

### Demographic and epidemiological characteristics

A total of 40 patients with clinical evidence for nocardiosis and one or more positive *Nocardia* cultures from various specimens were enrolled during the study period. Age, gender, underlying diseases and corresponding treatment are summarized in Table I.

The median age of patients was 52 (range, 28–82) years and 65.0% of the patients were male. A total of 72.5% (29/40) of the patients had one or more underlying diseases; diabetes (32.5%), autoimmune diseases (20.0%), chronic lung disease (15.0%), solid organ transplant (12.5%) and chronic renal disease (10.0%) were the most common underlying diseases. Five (12.5%) patients had diseases due to trauma-related injuries such as road traffic accidents and skin abrasion. Only one patient (2.5%) had HIV infection. The remaining five patients had no definable underlying diseases (12.5%). A total of 22 patients (55.0%) were found to be immunocompromised due to corticosteroids (20 patients, including seven patients on immunosuppressive therapy), chemotherapy (one patient) or HIV infection (one patient).

### Clinical features and laboratory abnormalities

The clinical features and laboratory abnormalities of the patients are shown in Table II. The clinical features of nocardiosis are generally non-specific. Fever, cough, the production of sputum and chest pain were the most common symptoms observed among the study participants. The majority of the patients presented a subacute form of infection with one patient having a chronic form of cutaneous abscess infection, and four patients presented an acute form of infection. Overall, the pleuropulmonary region (n=34, 85.0%) was the most involved site among the study population, which included the lung (n=29, 72.50%) and pleura (n=5, 12.5%). The other sites involved included the skin and soft tissue (25.0%), brain (12.5%), and pericardium (2.5%).

Disseminated disease, defined as the isolation of *Nocardia* from the bloodstream or its involvement in multiple organs, was demonstrated in 30.0% of cases. Within the disseminated disease group, ten patients (83.3%) had lung involvement, five (41.7%) had central nervous system (CNS) involvement, five (41.7%) had cutaneous abscess and one patient (8.3%) had suppurative pericarditis. Bacteremia was present in five patients, one each with renal transplantation, liver transplantation, autoimmune hemolytic anemia, diabetes mellitus, and one patient without underlying disease. The median time from the onset of symptoms to the diagnosis of nocardiosis was 42 (range, 9–120) days. In patients with and without underlying diseases, the median time from hospital admission to diagnosis was 53.8 and 12.1 days, respectively.

Leukocytosis (defined as a leukocyte count of >10×10^9^/l) was present in 21 (52.5%) patients. Neutrophilia (>75% neutrophils) was present in 30 (75.0%) patients. No patients had leukopenia (leukocyte count <4×10^9^/l) or neutropenia (neutrophil count <0.5×10^9^/l). C-reactive protein levels were available in 26 (65.0%) patients and they markedly increased (defined as a level >8 mg/l) in 19 (73.1%) patients with a range of 30.2 to 217 mg/l. Only three patients (7.5%) had a completely normal blood count and chemistry panel on admission.

Diagnostic cultures were obtained from the following clinical samples: sputum in 16 patients, bronchoalveolar lavage in one, empyema drainage in 10, lung biopsy in three, abscess puncture fluid in eight and blood in five patients.

Concomitant infections were commonly observed in the study participants. A total of 40.0% of patients with pulmonary nocardiosis were coinfected with other bacteria or fungi including *Stenotrophomonas maltophilia, Klebsiella pneumonia, Streptococcus pneumoniae, Enterococcus faecalis, Aspergillus* and *Candidiasis*. One patient with pleurisy was coinfected with *Escherichia coli* and another with *Pseudomonas aeruginosa*. One patient with a brain abscess had a *Nocardia* coinfection with *Pseudomonas aeruginosa*.

### Major findings on chest computed tomography (CT) and brain magnetic resonance imaging (MRI) scans

**Figure 1:**
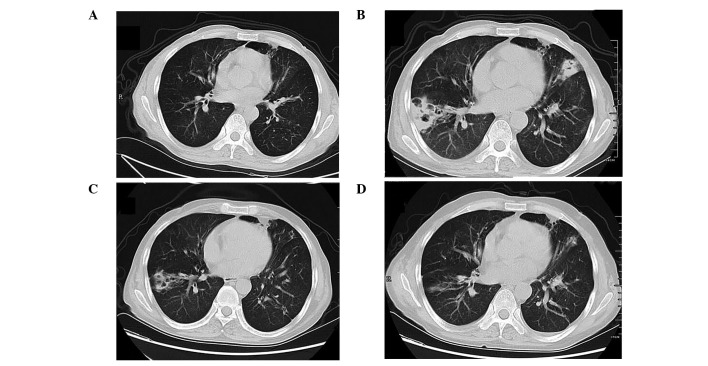
A chest computed tomography (CT) scan of a 47-year-old male with allergic pulmonary aspergillosis and pulmonary nocardiosis. Seven weeks prior to the onset of nocardiosis (A: 21/1/2013), the lung window image shows no notable lesions. On day eight following the onset of illness (B: 21/3/2013), the lung window image shows multiple new nodules with cavitation in both lungs. CT scans (C: 16/4/2013 and D: 28/5/2013) demonstrated clear improvements in inflammation following antibiotic therapy.

Chest X-ray results of pulmonary nocardiosis are usually non-specific and similar to those caused by other bacteria. A total of 36 patients had serial chest CT scans and commonly observed abnormalities were airspace opacities, nodules and masses, which accounted for 88.2% of cases. Cavitation was common in patients with pulmonary nocardiosis (47.1%; Fig. 1); alveolar and interstitial infiltration were less common. Pleural effusion or pleural nodules were found alone or concurrently with lung lesions.

Results of ^18^F-fluorodeoxyglucose (^18^F-FDG) positron emission tomography and CT scans were available for two (5%) patients (one with pulmonary nocardiosis and the other with a nocardial pleural nodule). An increased ^18^F-FDG uptake demonstrated in the lung and pleural nodules in these patients was consistent with a previous study (10). Chest pathology was observed in seven patients (17.5%) and tissue pathology tests revealed granulomatous inflammation or abscesses. The radiologic patterns were not specific.

**Figure 2:**
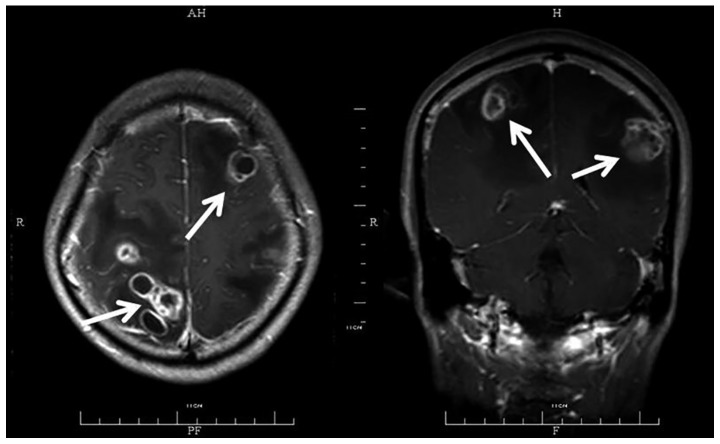
Contrast-enhanced magnetic resonance imaging scan of a 45-year-old male without any underlying diseases but with disseminated central nervous system nocardiosis, showing bilateral involvement of the brain. Multiple ring lesions with surrounding edema are shown (arrow), indicating multiple abscesses.

Five patients with brain nocardiosis had undergone brain CT or MRI scans and were found to have a solitary ring-enhancing lesion with central necrosis and surrounding edema within the corresponding localization, which was associated with abscesses (Fig. 2).

### Treatment and outcome

Two patients were referred to a local hospital immediately following diagnosis and thus were unavailable for follow-up. Therefore, details of antimicrobial therapy were only available for the remaining 38 patients.

A total of 27 (71.1 %) patients initially received treatment with trimethoprim sulfamethoxazole (TMP-SMX). Of these, four patients received TMP-SMX alone, while the others received it in combination with: carbapenem (eight patients), amikacin (two patients), ceftriaxone (six patients), quinolones (one patient), linezolid (three patients), or carbapenem and amikacin (three patients).

Three patients received treatment with quinolones either as a monotherapy (one patient) or in combination with minocycline or with TMP-SMX (one patient, respectively). The other antibiotic regimens included a combination of amikacin and carbapenem (one patient), ceftriaxone (four patients), carbapenem (one patient), linezolid (one patient) and a combination of amikacin, carbapenem and minocycline (one patient).

Following 2–4 weeks of initial treatment, the patients were discharged as their clinical status had improved. Their regimen was switched to the following oral treatments: TMP-SMX; linezolid; TMP-SMX in combination with linezolid; quinolones; TMP-SMX in combination with quinolones; and quinolones in combination with minocycline.

Six patients (15.0%) succumbed during hospitalization; of these, three had disseminated disease, two had severe pneumonia and one succumbed due to hepatitis B virus-related liver failure. Among the six deceased patients, a diagnosis of nocardiosis was missed for three patients. The mortality rate was higher among patients with disseminated disease (36.4%), immunocompromised patients (22.7%), elderly patients >55 years old (31.2%), patients who had CNS involvement (60.0%) and patients with concomitant infections (31.2%). There was no significant difference in mortality between males (19.2%) and females (14.3%). Follow-up details for >3 months were available for 29 patients (72.5%) and only 27 had follow-up details for one year. Other patients were referred to other hospitals and thus were unavailable for follow-up. Following hospital discharge, three patients succumbed due to underlying diseases (one each with lung cancer, renal failure and HIV infection), none of whom had CNS involvement. Two patients did not receive neurosurgery and one patient who received neurosurgery developed a concomitant infection with *Pseudomonas aeruginosa* in the brain abscess.

Primary cutaneous nocardiosis was treated in all patients. The duration of treatment ranged from one to three months and three patients received antibiotic therapy in combination with surgical treatment. One patient with disseminated disease involving the lung, pericardium and subcutaneous regions recovered following seven months of treatment, but recurrence of the disseminated disease occurred one year following the discontinuation of treatment. However, this patient recovered completely from the second disseminated disease.

The most common initial diagnoses were pulmonary fungal infection and tuberculosis. Due to the observation of multiple nodules with cavities, mass and pleural effusion in the CT scans at the time of initial presentation, a fungal infection was highly suspected. A total of 17 patients (42.5%) initially received antifungal therapy with voriconazole, caspofungin, amphotericin B or fluconazole. The two patients with brain involvement received praziquantel for antiparasitic treatment due to an initial diagnosis of parasitic infection.

## Discussion

The present retrospective study from a large tertiary teaching hospital is the first report on serial cases of nocardiosis in China. Similar to other reports, nocardiosis was observed to be an emerging disease. While only one patient was diagnosed with nocardiosis during 2000, approximately seven cases were reported during 2012 at the site of the current study. This increased trend is possibly due to the rise in the number of immunosuppressed patients over the last several years (11). The most common underlying disease in the patients with nocardiosis was diabetes (32.5%). Autoimmune disease (20.0%), bronchiectasis and other structural lung abnormalities, including chronic obstructive pulmonary disease (15.0%), and organ transplantation (12.5%), were also observed as risk factors in the study population. These observations were in accordance with other published studies (4–8). Notably, despite the hospital being a bone marrow transplantation (BMT) center, none of the patients were BMT recipients. In addition, the study site was in an area where HIV is not epidemic, and only one patient in the study had HIV infection. Nocardiosis in the study subjects had male predominance, which was similar to most previously published studies (4,6,8,12). The major risk factor in the study population was corticosteroid treatment, with 20 (50.0%) of the patients having received systemic corticosteroid therapy prior to the diagnosis of nocardiosis. Chronic corticosteroid treatment at a high dose has been reported to play a predisposing role in nocardiosis (2,3,6,13). However, the present study results revealed that even short-term corticosteroid treatment at a low dose (0.5–1 mg/kg, 3–6 weeks duration) may be a risk factor for nocardiosis.

As reported in other studies (2,3,6–8), pulmonary disease was the most common presentation (85.0%) and 30.0% of cases had a disseminated disease. The most frequent chest CT results in the study group were airspace opacities, multifocal nodules or masses; cavitation was common in pulmonary nocardiosis. The brain images demonstrated lesions associated with abscesses.

Clinical recognition of nocardial infection is difficult due to its relatively low incidence rate and non-specific manifestations. In the current study, the common initial diagnosis was general bacterial infection, invasive fungal disease, tuberculosis, parasitic infection or malignancy. In three patients, an official diagnosis of nocardiosis was established only following mortality. The median time between the onset of symptoms and diagnosis was 42 days, and such a delay was also reported in another published study (12). This delay in diagnosis and treatment may be life threatening. Primary pulmonary infection and skin/soft tissue infection are the most common initial clinical symptoms and may result in disseminated *Nocardia* infection if the initial therapy is inadequate. In the present study, at least 50% of disseminated nocardiosis cases were initially treated with antibiotics which is sensitive to *Nocardia*, such as carbapenem, amikacin, ceftriaxone or quinolones for suspected common bacterial infection. Such antibiotic treatment was discontinued due to the initial clinical improvement. However, the duration was not sufficient for nocardiosis and resulted in a tendency to disseminate. To reduce the delay in diagnosis and treatment, nocardiosis should be considered in the differential diagnosis of immunosuppressed patients and testing for *Nocardia* should be performed in patients with risk factors for nocardiosis.

To the best of our knowledge, there have been no randomized prospective trials published to date that have assessed the most effective antimicrobial therapy for *Nocardia* infection. In the present study, 71.1% of patients who received antimicrobial agents were treated with TMP-SMX monotherapy or a combination of TMP-SMX with other drugs. Alternative antibiotic regimens included the combination of amikacin and carbapenem, ceftriaxone, linezolid, quinolones, and minocycline.

TMP-SMX is currently accepted as the first-line treatment for nocardiosis, although there are controversial reports available concerning the sensitivity of TMP-SMX to *Nocardia*. Certain studies have revealed high rates of resistance (16.1–43.0%) of *Nocardia* to TMP-SMX (6,14,15). However, a study by Brown-Elliott *et al* involving 522 clinical isolates in six major laboratories in the United States reported that only 2% of the isolates were demonstrated to have resistant minimum inhibitory concentrations of TMP-SMX and/or SMX (16). Data from a study by Lai *et al* revealed that 10% of isolates were resistant to TMP-SMX (17). Furthermore, the antimicrobial susceptibility of *Nocardia* to meropenem, amikacin and ceftriaxone has also been reported to be high (17). Clinical experience with these drugs proved encouraging (18,19). The mortality rate remains high with TMP-SMX monotherapy, especially in patients with severe or disseminated disease. Thus, a combination of TMP-SMX with amikacin and imipenem or ceftriaxone is recommended as an empirical therapy in serious CNS-involved and disseminated cases of *Nocardia*. Regimens with quinolones have been described in several case reports, especially in patients with CNS nocardiosis, due to their excellent cerebrospinal fluid penetration and low toxicity (20,21). Minocycline is another potentially useful drug (22,23) whilst linezolid has been indicated to be an effective alternative for the treatment of nocardiosis in certain studies (5,10,23–26). Previous clinical experience obtained from the study site has revealed that linezolid has a marked effectiveness in treating *Nocardia* infection (24,26). However, long-term use of linezolid should be avoided due to the cost factor and adverse effects. Treatment for nocardiosis is recommended to begin at the initial diagnosis, continue for several weeks intravenously and then switch to oral therapy following initial clinical improvement. For local empyema, including thoracic empyema, brain and cutaneous abscesses, adjuvant surgical treatment may be necessary.

The following were the key limitations of the present study: i) the study was retrospective and ii) the majority of the microbiological data concerning the identification of species and their susceptibility were unavailable. Despite these limitations, the current study results provide useful information on the risk factors of nocardiosis. *Nocardia* infection is an uncommon but emerging disease that may cause serious or disseminated disease. Delays in the diagnosis and treatment of nocardiosis may be life threatening. Patients with nocardiosis, especially those who are at high risk, require careful clinical and pathological evaluations. Early recognition of the disease and the initiation of appropriate treatment are essential for a good prognosis.

## Figures and Tables

**Table I:** Demographic and epidemiological characteristics of 40 patients with nocardiosis.

Characteristic	Number	%
Mean age (years)	52.2	100.0
Gender distribution		
Male	26	65.0
Female	14	35.0
Underlying disease		
Transplantation	5	12.5
Liver	3	7.5
Kidney	2	5.0
Chronic lung disease	6	15.0
Solid malignancy	2	5.0
Diabetes	13	32.5
Hypertension	4	10.0
Autoimmune diseases	8	20.0
Chronic renal disease	4	10.0
Hematological disease	3	7.5
Hepatitis B infection	3	7.5
HIV infection	1	2.5
Chronic drug use		
Corticosteroids	20	50.0
Chemotherapy	1	2.5
Immunosuppressants	7	17.5
Trauma	5	12.5
Other	2	5.0

Autoimmune diseases (n): systemic lupus erythematosus (3), dermatomyositis (3), rheumatoid arthritis (1), uveitis (1). Hematological diseases: aplastic anemia (1), autoimmune hemolytic anemia (2). Solid malignancy: thymoma (1), lung cancer (1). Other: rheumatic valvular heart disease (1) HIV, human immunodeficiency virus.

**Table II:** Clinical characteristics and laboratory abnormalities of 40 patients with nocardiosis.

Characteristics	Number	%
Clinical feature		
Fever	30	75.0
Cough	26	65.0
Sputum production	20	50.0
Chest pain	9	22.5
Hemoptysis	3	7.5
Cutaneous abscess/ulcer	5	12.5
Shock	1	2.5
Headache	4	10.0
Laboratory abnormalities		
Leukocytosis	21	52.5
Neutrophilia	30	75.0
C-reactive protein >8 mg/l	19	73.1

## References

[1] Wilson JW (2012). Nocardiosis: updates and clinical overview. Mayo Clin Proc.

[2] Rosman Y, Grossman E, Keller N, Thaler M, Eviatar T, Hoffman C, Apter S (2013). Nocardiosis: A 15-year experience in a tertiary medical center in Israel. Eur J Intern Med.

[3] Cattaneo C, Antoniazzi F, Caira M, Castagnola C, Delia M, Tumbarello M (2013). *Nocardia spp* infections among hematological patients: results of a retrospective multicenter study. Int J Infect Dis.

[4] Garcia-Bellmunt L, Sibila O, Solanes I, Sanchez-Reus F, Plaza V (2012). Pulmonary nocardiosis in patients with COPD: characteristics and prognostic factors. Arch Bronconeumol.

[5] Ambrosioni J, Lew D, Garbino J (2010). Nocardiosis: updated clinical review and experience at a tertiary center. Infection.

[6] Mootsikapun P, Intarapoka B, Liawnoraset W (2005). Nocardiosis in Srinagarind Hospital, Thailand: review of 70 cases from 1996–2001. Int J Infect Dis.

[7] Saubolle MA, Sussland D (2003). Nocardiosis: review of clinical and laboratory experience. J Clin Microbiol.

[8] Castro JG, Espinoza L (2007). *Nocardia* species infections in a large county hospital in Miami: 6 years experience. J Infec.

[9] Tuo MH, Tsai YH, Tseng HK, Wang WS, Liu CP, Lee CM (2008). Clinical experiences of pulmonary and bloodstream nocardiosis in two tertiary care hospitals in northern Taiwan, 2000–2004. J Microbiol Immunol Infect.

[10] Zhao K, Dong MJ, Sheng ZK, Liu KF, Yang SY, Liu ZF, Sheng JF (2012). Elevated uptake of ^18^F-FDG in PET/CT imaging of a nocardial pleural nodule. Clin Imaging.

[11] Hannaman MJ, Ertl MJ (2013). Patients with immunodeficiency. Med Clin North Am.

[12] Matulionyte R, Rohner P, Uckay I, Lew D, Garbino J (2004). Secular trends of nocardia infection over 15 years in a tertiary care hospital. J Clin Pathol.

[13] Martínez Tomás R, Menéndez Villanueva R, Reyes Calzada S, Santos Durantez M, Vallés Tarazona JM, Modesto Alapont M, Gobernado Serrano M (2007). Pulmonary nocardiosis: risk factors and outcomes. Respirology.

[14] Uhde KB, Pathak S, McCullum I, Jannat-Khah DP, Shadomy SV, Dykewicz CA (2010). Antimicrobial-resistant *Nocardia* isolates, United States, 1995–2004. Clin Infect Dis.

[15] Tremblay J, Thibert L, Alarie I, Valiquette L, Pépin J (2011). Nocardiosis in Quebec, Canada, 1988–2008. Clin Microbiol Infect.

[16] Brown-Elliott BA, Biehle J, Conville PS, Cohen S, Saubolle M, Sussland D (2012). Sulfonamide resistance in isolates of *Nocardia* spp. from a US multicenter survey. J Clin Microbiol.

[17] Lai CC, Liu WL, Ko WC, Chen YH, Tan HR, Huang YT, Hsueh PR (2011). Multicenter study in Taiwan of the in vitro activities of nemonoxacin, tigecycline, doripenem, and other antimicrobial agents against clinical isolates of various *Nocardia* species. Antimicrob Agents Chemother.

[18] Garcia del Palacio JI, Martín Pérez I (1993). Response of pulmonary nocardiosis to ceftriaxone in a patient with AIDS. Chest.

[19] Lo W, Rolston KV (1993). Use of imipenem in the treatment of pulmonary nocardiosis. Chest.

[20] Fihman V, Berçot B, Mateo J, Losser MR, Raskine L, Riahi J (2006). First successful treatment of *Nocardia farcinica* brain abscess with moxifloxacin. J Infect.

[21] Tanioka K, Nagao M, Yamamoto M, Matsumura Y, Tabu H, Matsushima A (2012). Disseminated *Nocardia farcinica* infection in a patient with myasthenia gravis successfully treated by linezolid: a case report and literature review. J Infect Chemother.

[22] Ogawa T, Kasahara K, Yonekawa S, Nakagawa C, Maeda K, Konishi M (2011). *Nocardia beijingensis* pulmonary infection successfully treated with intravenous β-lactam antibiotics and oral minocycline. J Infect Chemother.

[23] Lewis KE, Ebden P, Wooster SL, Rees J, Harrison GA (2003). Multi-system infection with *Nocardia farcinica*-therapy with linezolid and minocycline. J Infect.

[24] Shen Q, Zhou H, Li H, Zhou J (2011). Linezolid combined with trimethoprim-sulfamethoxazole therapy for the treatment of disseminated nocardiosis. J Med Microbiol.

[25] Yu X, Han F, Wu J, He Q, Peng W, Wang Y (2011). *Nocardia* infection in kidney transplant recipients: case report and analysis of 66 published cases. Transpl Infect Dis.

[26] Shen T, Wu L, Geng L, Wei Z, Zheng S (2011). Successful treatment of pulmonary *Nocardia farcinica* infection with linezolid: case report and literature review. Braz J Infect Dis.

